# Growth Hormone (GH) Deficient Mice With GHRH Gene Ablation Are Severely Deficient in Vaccine and Immune Responses Against *Streptococcus pneumoniae*

**DOI:** 10.3389/fimmu.2018.02175

**Published:** 2018-10-02

**Authors:** Khalil Farhat, Gwennaëlle Bodart, Chantal Charlet-Renard, Christophe J. Desmet, Michel Moutschen, Yves Beguin, Frédéric Baron, Pierrette Melin, Pascale Quatresooz, Anne-Simone Parent, Daniel Desmecht, Jean-Claude Sirard, Roberto Salvatori, Henri Martens, Vincent G. Geenen

**Affiliations:** ^1^GIGA-I3 Center of Immunoendocrinology, University of Liège, Liège, Belgium; ^2^GIGA-I3 Cellular and Molecular Immunology, University of Liège, Liège, Belgium; ^3^GIGA-I3 Infectious diseases, University of Liège, Liège, Belgium; ^4^GIGA-I3 Hematology, University of Liège, Liège, Belgium; ^5^Department of Clinical Microbiology, University Hospital of Liège, University of Liège, Liège, Belgium; ^6^Department of Human Histology, University of Liège, Liège, Belgium; ^7^Division of Pediatric Endocrinology, University Hospital of Liège, Liège, Belgium; ^8^Department of Veterinary Pathology, University of Liège, Liège, Belgium; ^9^CNRS, INSERM, CHU Lille, Institut Pasteur de Lille, U1019-UMR8204–Center for Infection and Immunity of Lille, University of Lille, Lille, France; ^10^Johns Hopkins University School of Medicine, Baltimore, MD, United States

**Keywords:** somatotrope axis, GHRH, GH, *S. pneumonia*, thymo-independent antigen, spleen

## Abstract

The precise impact of the somatotrope axis upon the immune system is still highly debated. We have previously shown that mice with generalized ablation of growth hormone (GH) releasing hormone (GHRH) gene (*Ghrh*^−/−^) have normal thymus and T-cell development, but present a marked spleen atrophy and B-cell lymphopenia. Therefore, in this paper we have investigated vaccinal and anti-infectious responses of *Ghrh*^−/−^ mice against *S. pneumoniae*, a pathogen carrying T-independent antigens. *Ghrh*^−/−^ mice were unable to trigger production of specific IgM after vaccination with either native pneumococcal polysaccharides (PPS, PPV23) or protein-PPS conjugate (PCV13). GH supplementation of *Ghrh*^−/−^ mice restored IgM response to PPV23 vaccine but not to PCV13 suggesting that GH could exert a specific impact on the spleen marginal zone that is strongly implicated in T-independent response against pneumococcal polysaccharides. As expected, after administration of low dose of *S. pneumoniae*, wild type (WT) completely cleared bacteria after 24 h. In marked contrast, *Ghrh*^−/−^ mice exhibited a dramatic susceptibility to *S. pneumoniae* infection with a time-dependent increase in lung bacterial load and a lethal bacteraemia already after 24 h. Lungs of infected *Ghrh*^−/−^ mice were massively infiltrated by inflammatory macrophages and neutrophils, while lung B cells were markedly decreased. The inflammatory transcripts signature was significantly elevated in *Ghrh*^−/−^ mice. In this animal model, the somatotrope GHRH/GH/IGF1 axis plays a vital and unsuspected role in vaccine and immunological defense against *S. pneumoniae*.

## Introduction

In the framework of intimate interactions between immune and neuroendocrine systems, growth hormone (GH) has been proposed to exert regulatory effects on the immune system, by binding to and activating GH receptor (GHR). This receptor belongs to type I cytokine receptors, and is present on cell surface of many immune cells, such as natural killer (NK) cells, B cells, T cells, monocytes and thymic epithelial cells (TEC) both in humans and mice (1–4). GH regulates adhesion and migration of neutrophils, monocytes and macrophages at the site of inflammation (5–7), enhances production of IgM and IgG antibodies by human tonsillar B cells (8), and increases T cell proliferation in cultured mouse splenocytes (9). GH is also involved in regulation of human T and B cell apoptosis, protecting them from irradiation-induced cell death by enhancing expression of the anti-apoptotic protein, B-cell lymphoma 2 (Bcl-2) (10). Dorshkind and Horseman suggested that GH could play an immunoprotective role by counteracting negative immunoregulatory signals, such as glucocorticoid levels that increase during stressful conditions (11, 12).

In order to further define the physiological impact of somatotrope axis upon the immune system, we used a knockout (KO) mouse model resulting from a targeted generalized disruption of growth hormone-releasing hormone (GHRH) gene, which results in a dwarf phenotype due to severe GH deficiency (13). We have previously reported that *Ghrh*^−/−^ mice exhibit normal thymic function and T cell development, but have a severe spleen atrophy and a decrease in B cell percentage when compared to their wild-type (WT) littermates (14). These observations prompted us to investigate the immune response of *Ghrh*^−/−^ mice against T-cell independent type 2 antigens (TI-2), such as *Streptococcus pneumoniae* (*S. pneumoniae*) against which immune response is mainly based on B cells and antibody secretion. *S. pneumoniae* is an encapsulated gram-positive bacterium responsible of pneumonia and meningitis, particularly in neonates and adults above 50 year old (15). Anti-capsular polysaccharide antibodies play an important role in the protection against these pathogens and current pneumococcal vaccines are composed of pneumococcal capsular polysaccharides from serotypes mostly involved in invasive diseases. Two pneumococcal vaccines are currently used: one is a polysaccharide vaccine (PPV23), which covers 23 pneumococcal serotypes, and primarily induces a B cell dependent response in the absence of major histocompatibility complex II-restricted T cell help (16, 17), and hence referred to as TI-2 antigens (18). PPV23 stimulates B-1 cells and splenic marginal zone (MZ) to produce anti-capsular antibodies (19). The other commonly used vaccine is 13-valent pneumococcal conjugate vaccine (PCV13), composed of 13 pneumococcal serotypes most frequently involved in invasive infection, which elicit antibody isotype switching, stimulation of follicular B cell region and conversion of the capsular polysaccharide into a T cell dependent antigen (20, 21). In addition to anti-capsular antibodies, innate immunity plays an important role in the protection against *S. pneumoniae* respiratory infection by early recruitment of inflammatory cells, in particular neutrophils (22, 23). Activation and recruitment of alveolar macrophages constitutes another key element of innate immunity by playing a crucial role in phagocytosis, inflammatory cytokine secretion, and antigen presentation (24). Finally, the activation of classical complement pathway by IgM has been shown to play an important role in protection against bacteraemia during pneumococcal respiratory infection (25).

Consequently in this study, we investigated the responses of *Ghrh*^−/−^ mice to anti-*S. pneumoniae* vaccines, and we set up an animal protocol which consist to test a non-lethal *S. pneumoniae* dose, defined by full clearance by WT mice 24 h post-infection (26). For this purpose, mice were inoculated by *S. pneumoniae* via intra-nasal (i.n.) route, and immune response was evaluated by quantifying bacterial load in lung homogenates and blood, measuring the percentage of immune cells recruited to the site of infection, by histological and RT-PCR analysis.

## Animals and methods

### Animals

*Ghrh*^−/−^ (mouse strain C57BL6/j background) was previously described (13). Wild-type (WT) C57BL/6j mice were purchased from Charles River Laboratories. All animals were bred in ventilated cages at the Central Animal Facility of Liege University (GLP certified, LA.2610359) with a 12 h light/12 h dark cycle and controlled conditions of humidity and temperature with food and water *ad libitum*. We performed backcross between those two strains, in order to obtain animals with completely identical genetic background. Briefly, *Ghrh*^−/−^ and C57Bl/6 mice were bred together to obtain a F1 generation of heterozygous (HZ) animals. F1 animals were mated together and gave rise to F2 mice with *Ghrh*^+/+^ (called WT in the text), *Ghrh*^+/−^ (HZ) and *Ghrh*^−/−^ (referred as KO in the text) animals (respectively 25–50–25% proportion expected). Mouse genotype was identified phenotypically: because the agouti color (a dominant trait) is located near the *Ghrh* gene (and the ES cells used to generate these mice were from agouti color 129SV mice) the presence of the ablated *Ghrh* allele can be followed looking at fur color: WT backcrossed F2 mice are black and normal-sized; HZ backcrossed animals are agouti and normal-sized, and KO backcrossed mice are agouti and dwarf. Normal-sized and dwarf mice were separated at least 4 weeks before any experiment. Male and female mice of 3 months were used for characterization experiments, and for GH supplementation experiments. All experiments were conducted with approval of the Institutional Animal Care and Use Committee of the University of Liège (permit n°1805) in strict accordance with guidelines for the care and use animals set out by the European Union.

### Vaccination

WT and KO mice were immunized by subcutaneous (s.c) administration of PPV23 vaccine (Pneumovax 23®, serotypes 1, 2, 3, 4, 5, 6B, 7F, 8, 9N, 9V, 10A, 11A, 12F, 14, 15B, 17F, 18C, 19F, 19A, 20, 22F, 23F, 33F) or PCV13 conjugate vaccine (Prevnar 13®, serotypes 1, 3, 4, 5, 6A, 6B, 7F, 9V, 14, 18C, 19A, 19F, and 23F) at days 0 and 21. Blood samples were collected prior to administration of the primary immunization dose at day 0.

For dose response study, mice were immunized with two equal doses (at days 0 and 21) consisting of either 0.1, 1.1, or 2.2 μg/vaccine serotype (except for serotype 6B, which was doubled in concentration) diluted in sterile Dulbecco's Phosphate Buffered Saline (DPBS) and injected in a final volume of 200 μl. The 2.2 μg/serotype dose exhibited best immune response and was chosen for following vaccine experiments (data not shown).

Every week, 8 mice/group were bled using a tail bleeding assay under infrared light, and samples were individually examined for IgM antibody levels by Enzyme-Linked Immunosorbent Assay (ELISA).

For supplementation study, *Ghrh*^−/−^ groups were supplemented by intraperitoneal (i.p.) injection of recombinant human GH (hGH, 1 mg/kg in 100 μl DPBS, Genotonorm, Pfizer) or DPBS (vehicle control) for 5 weeks:2 weeks before and 3 weeks after first vaccination at day 0. Glycemia and weight were measured in order to follow the effect of hGH treatment after each sampling.

### IgM ELISA

In order to detect specific IgM antibodies against pneumococcal polysaccharide type 1 (PnPS1), ELISA was performed as described previously (27) by using both cell wall polysaccharide (CWPS) and 22F polysaccharide absorption with a slight modification regarding incubation time. Briefly, 96-well plates (Thermofisher ref 469949) were coated with 1 μg/well of PnPS1 (Statens Serum Institut, Copenhagen, Denmark) at 4°C overnight, in a humidified chamber. Plates were then washed with a washing buffer (0.01 M PBS, 0.1% tween), and blocked with 300 μl/well of blocking solution [PBS 1X, 1% bovine serum albumin (BSA, RIA grade, VWR A0850), 5 μg/ml 22F capsular polysaccharide and CWPS (Statens Serum Institut, Copenhagen, Denmark), for 2 h at 37°C with gentle agitation. Test sera were diluted to 1:400 in incubation buffer (PBS 1X, 0.5% BSA, 0.1% tween, 5 μg/ml 22F capsular polysaccharide and CWPS) and incubated for 20 min at room temperature (RT). Each sample (100 μl/well) was transferred to coated microtiter plate and incubated for 2 h at 37°C with gentle agitation. After washing step, 100 μl of diluted horseradish peroxidase-coupled goat anti-mouse IgM (Gentaur, Star86P) were added in each well and plates were incubated for 1 h at 37°C with gentle agitation. Finally, 100 μl/well of 3,3′,5,5′-tetramethyl-benzidine (TMB, Invitrogen), were added and incubated in dark at RT with gentle agitation. The reaction was stopped after 20 min by addition of 25 μl/well of sulphuric acid. Optical density (OD) was read at 450 nm with a Victor Multilabel Plate Reader (Victor X3, PerkinElmer, USA). In order to normalize ELISA results, we chose from dose response study of PCV13 a reference serum that was considered as a positive control by presenting the best IgM immune response and was assigned at 100% arbitrary unit (a.u). The relative percentage of each sample was calculated in comparison to this running positive control.

### Bacterial strains and cultures

*S. pneumoniae* serotype 1 (clinical isolate E1586) sequence type ST304 (28) was kindly provided by Dr Jean-Claude Sirard (Pasteur Institute, Lille, France) and was used for all experiments. Working stocks (23, 26) were prepared by inoculating fresh *S. pneumoniae* colonies (cultured overnight at 37°C in 5% CO_2_ on blood-agar plates) in Todd Hewitt Yeast Broth medium (THYB, Sigma-Aldrich) further incubated at 37°C to reach an OD of 0.5 at 600 nm. Cultures were then stored at −80°C in THYB + glycerol 10% (v/v), as single-use aliquots up to 3 months. Before and after thawing, bacterial viability and enumeration in stock aliquots were performed by plating serial dilutions of bacterial suspension onto blood agar incubated overnight at 37°C and 5% CO_2_ onto blood agar. *S. pneumoniae* colonies were discriminated by typical green halo caused by its alpha-hemolytic activity. After stock, presence of other microbial contamination was checked by thawing last aliquot and inoculate ~10 microliters onto a blood agar plate. After overnight growth at 37°C, only alpha-hemolytic pneumococcal colonies were visible. The sensibility of the stock to optochin antibiotics at 0.5X assay concentrations was confirmed after stock preparation.

### *S. pneumoniae* infection

Mice were anesthetized by i.p. injection of Ketamine (80 mg/kg) and Xylazine (10 mg/kg). Frozen bacterial aliquots were thawed, centrifuged for 5 min at 2,500 × g, washed and suspended in sterile DPBS to reach appropriate concentration. The sublethal dose was defined as 4 × 10^4^ colony forming units (CFU) per mouse (26). For infection, this dose of *S. pneumoniae* or DPBS (control solvent) were inoculated in 30 μl via i.n. route. Mice survival was daily recorded, and euthanized upon showing signs of severe morbidity (>3, Supplementary Table 1). Mice were sacrificed at 0, 6, 24, and 48 h post-infection, by i.p. injection of Ketamine (80 mg/kg) and Xylazine (10 mg/kg followed by cardiac puncture. Lungs, spleen and blood were collected for further analysis.

### Determination of lung bacterial load

Infected whole lungs were collected from sacrified mice, transported in sterile DPBS and homogenized by pestles and cordless motor for pellet mix (VWR 431-0100). Serial dilutions of each homogenate were plated onto blood-agar plates (VWR, 100253ZFMP) to enumerate CFU of *S. pneumoniae* and to determine the level of infection. *S. pneumoniae* colonies were discriminated by typical green halo caused by its alpha-hemolytic activity. As a surrogate marker of bacteremia, detection of bacteria was also assayed in the spleen, by plating spleen homogenate onto blood-agar.

### Histology

Infected and non-infected lungs and spleens were fixed with 4% paraformaldehyde (PFA) at 4°C overnight, and were embedded in paraffin. Tissues were sectioned at 6 μm, mounted on Superfrost glass slides (Fischer Scientific) and stained with Hematoxylin-Eosin (HE, Merck millipore, 1.05174.1000, 1.09844.1000). Slides were imaged with FSX100 Inverted Microscope (Olympus).

### Flow cytometry

For immune cell subtype analysis, lungs and spleens from infected and non-infected mice were collected in RPMI (VWR, LONZ17-512F) supplemented with 10% Fetal Bovine Serum (FBS, Thermofisher, 10500056) and 1% of penicillin and streptomycin (P/S, Lonza, 2219), and homogenized by using cell dissociation sieve-tissue grinder kit (Sigma–Aldrich, CD1-1KT). Lung and spleen cells were isolated after collagenase Ia (2 mg/mL, Roche, 10103586001) and DNase I (1/250 dilution, Roche, 11284932001) treatment, separated with 20% percoll (GE healthcare Percoll, 17-0891-01), filtered through a 70 μm Nylon cell strainer (Miltenyibiotec, 130-110-916) and incubated on ice for 5 min, in 1 ml of RBC lysis Buffer Hybri-Max (ebioscience, 00-4300-54) to lyse red blood cells.

Cells were then counted in a Neubauer Chamber and 1 × 10^6^ cells were used for flow cytometry analysis. Briefly, cells were washed with FACS buffer (DPBS, 2%FBS, 2 mM EDTA), blocked with Fc block (1 μg/ml, ebioscience, 14.0161.81) for 20 min and stained with a cocktail of cell surface monoclonal antibodies (mAbs) diluted in FACS buffer: Anti-mouse CD45.2-FITC (clone 104, 561.874), CD3e-PE (clone 145-2C11, 553.063), Ly6G-PE/Cy7 (clone IA8, 560.601), CD11b-BV421 (clone M1/70, 562.605) and CD19-BV510 (clone 1D3, 562.956), were purchased from BD Biosciences. Anti-mouse F4/80-APC (clone BM8, 17-4801-80) were purchased from eBioscience. After 20 min of incubation at 4°C in dark, labeled cells were washed in FACS buffer and resuspended in DPBS prior to FACS analysis (FACS Verse, BD Biosciences).

For B cell subtype analysis, spleen from non-infected mice were collected in RPMI (supplemented with 10% FBS) and homogenized in cell dissociation sieve-tissue grinder kit (Sigma–Aldrich, CD1-1KT). Splenocytes were filtered through a 70 μm Nylon cell strainer (Miltenyibiotec, 130-110-916) and incubated on ice for 5 min, in 1 ml of RBC lysis Buffer Hybri-Max (ebioscience, 00-4300-54), to lyse red blood cells. Cells (5 × 10^5^) were then stained for 20 min at 4°C in dark, with the following mAbs diluted in FACS buffer: anti-mouse CD11b-BV421 (clone M1/70, 562.605) and CD5-BV510 (clone 53-7.3, 563.069) were purchased from BD Biosciences, anti-mouse IgM-PE (clone II/41, 12-5790-81) were purchased from eBioscience, anti-mouse CD21/CD35-PE/Cy7 (clone 7^E^9, 123420) were purchased from Biolegend, anti-mouse B220-APC (clone RA3-6B2, 561.880) and CD23-FITC (clone B3B4, 561.772) were purchased from BD pharmingen. Labeled cells were then washed in FACS buffer and suspended in DPBS prior to FACS analysis (FACS Verse, BD Biosciences).

### RT-qPCR

Lungs were collected and directly stored at −80°C in RNAlater (Qiagen, 76104) until RNA extraction. RNAlater solution was discarded and whole lungs were homogenized in lysis reagent (Qiagen, 79306) by using an Ultra-Turrax. RNA extraction was performed using NucleoSpin® RNA kit (Macherey-Nagel, 740955.10) according to manufacturer instructions. After extraction, RNA concentration was measured by NanoDrop ND-1000 (Thermo Scientific) and 250 ng were used for reverse-transcription using Transcriptor first strand cDNA synthesis Kit (Roche, 4896866001). To measure mRNA levels specific for mouse genes: *Il10, Ifng, Cxcl9* and *Cd40*, a quantitative PCR was performed in an iCycler (Biorad) using mix (Roche, 10814270001) and SYBR® Green probes (Eurogentec) in the following conditions: 5 min of initial denaturation at 95°C; 40 cycles of amplification at 95°C for 10 s; 60°C for 15 s; 72°C for 10 s; cooling at 40°C. Mouse *Hprt* was used as a house-keeping gene for the following genes *Il10, Ifng, Cxcl9 and Cd40*. For mouse transcripts *Il17a, Il22, Il6, Il1b, Cxcl2, Ccl20, and Csf3, a* quantitative PCR was performed using QuantiTect® SYBR® Green PCR Kit (Qiagen) according to supplier information in a 7900HT System (Applied Biosystems). Mouse *Actb* was used as a house-keeping gene for the remaining genes. The forward and reverse primer sequences are presented in Supplementary Table 2. The relative mRNA amount in each sample was calculated using the 2^−ΔΔCt^ method where ΔCt = Ct_gene of interest_ – Ct_house keeping gene_, and expressed as relative mRNA expression in infected group compared to their respective mock mice (29).

### Sera protein detection

Sera from sacrified mice were collected at specific post-infection time points (0, 6, 24, and 48 h) in EDTA-coated tubes treated, centrifuged and stored at −80°C until analysis. The concentration of different sera proteins was measured according to manufacturer's instructions: Albumin (OSR6102), α-antitrypsin (OSR6163), complement component C3 (C3, OSR6159), C-reactive protein (OSR6199), ferritin (OSR61203), IgA (OSR61171), IgM (OSR61173), and transferrin (OSR6152) were purchased from Beckman Coulter, USA. Samples were analyzed by using multi-assay AU480 Chemistry analyzer (Beckman Coulter, United States).

### H1N1 infection

The porcine influenza virus (H1N1) (A/swine/Iowa/4/76) was kindly provided by Daniel Desmecht. Viruses were adapted to mice by lung-to-lung passaging (30). Mice were anesthetized by i.p. injection of Ketamine (80 mg/kg) and Xylazine (10 mg/kg). Frozen virus aliquots were thawed, centrifuged for 5 min at 2,500 g, washed and diluted to a non-lethal dose in C57BL6 strain [9 plaque forming unit (pfu)] in sterile DPBS (31). For infection, 30 μl of H1N1 virus or DPBS were inoculated via i.n. route. Mice survival was daily recorded for body weights and temperature and euthanized when showing signs of morbidity (>3, Supplementary Table 1).

### Immunohistochemistry

The spleen of infected and non-infected mice was collected and frozen in tissue-Tek O.C.T freezing medium at −80°C until analysis. Tissues were sectioned at 6 μm, mounted on Superfrost+ glass slides (Fischer Scientific). Splenic sections were fixed in cold solution (−20°C) of 50% acetone (VWR, 20065.293) and 50% methanol (Millipore, 1.06009.5000) for 5 min, then incubated with protein serum free (DAKO, X0909) for 30 min at RT. The sections were then incubated overnight in dark wet room with 100 μl of an anti-macrophage scavenger receptor (MARCO) mAb (ED31, T-2026, BMA Biomedicals, Switzerland) solution (2 μl/mL), followed by a 30 min incubation at RT with 100 μl of a polyclonal rabbit anti-rat Immunoglobulins (ab 6703, abcam, UK) solution (4 μg/mL). Positive staining were revealed using goat anti-rabbit IgG (H+L) Alexa fluor 488 conjugate (A11034, Invitrogen, Belgium), incubated for 2 h at RT. All antibodies were diluted by DAKO real antibody diluent (S2022, DAKO). Slides were washed 3 times for 5 min after each step by distilled water, mounted with ProLong® Gold Antifade Mountant (P10144, Thermofisher, Belgium), viewed and photographed with an Olympus FSX100 inverted microscope.

### Statistical analyses

Statistical analyses were performed on Prism 4.0 software (GraphPad). Kolmogorov-Smirnov and Shapiro-Wilk normality tests were performed to evaluate Gaussian distribution of results. Unpaired *t*-test was applied when Gaussian distribution was verified, and Mann-Whitney test for non-Gaussian distributions. For multi-parametric analysis of GH supplementation, two-way ANOVA with Bonferroni post-test was used. Testing infection susceptibility of strains was performed with the Fisher-Yates exact test.

## Results

### *Ghrh^−/−^* mice are unable to trigger a specific vaccine response against *S. pneumoniae*

**Figure 1:**
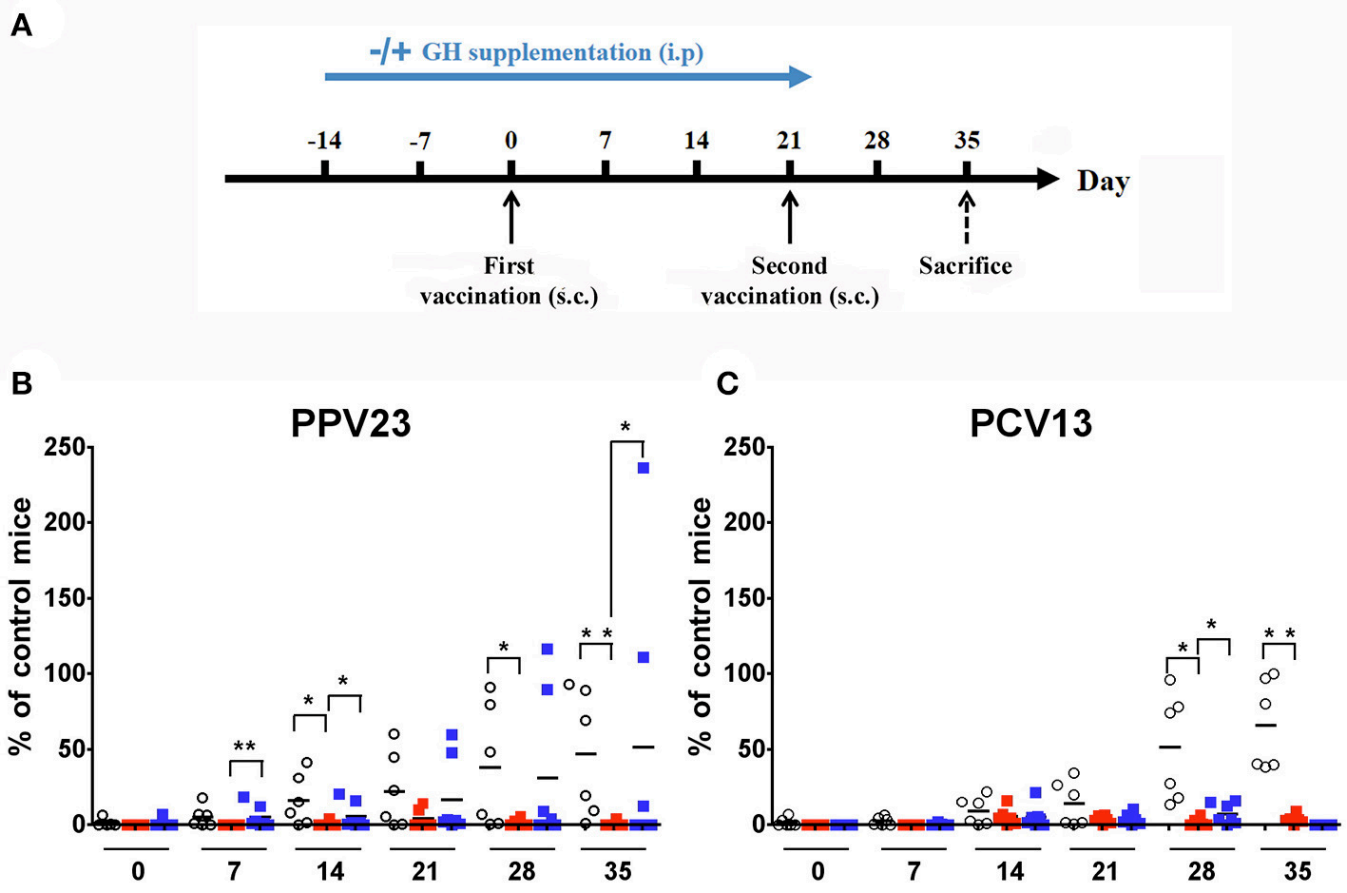
Vaccinal response to *S. pneumoniae* vaccines. **(A)** Experimental protocol for *S. pneumoniae* vaccination and hGH supplementation. KO and WT mice were immunized with PPV23 or PCV13 vaccine at days 0 and 21. HGH was supplemented to the *Ghrh*^−/−^ mice for 5 weeks (2 weeks before and 3 weeks after the first vaccination). The animals were bled prior to administration of the immunization dose and each week at day 0, 7, 14, 21, 28 and 35. s.c., subcutaneous. **(B,C)** Percentage of IgM antibodies from WT (◦), unsupplemented KO (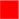) and supplemented KO mice (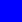) related to internal control. All results are presented as individual response and mean. *N* = 6 mice per group. *p < 0.05; **p < 0.001 (Mann-Whitney *U*-test).

We first investigated humoral response in WT and KO mice vaccinated with PPV23 or PCV13 by blood sampling and IgM measurements at time points shown in Figure 1A. We used three serial concentrations of vaccines without adaptation to mice weight as others have done (32). In preliminary experiments, the highest dose of both vaccine (2.2 μg/serotypes) was necessary to elicit a significant antibody level in WT mice. The lower doses (1.1 and 0.1 μg/serotypes) only induced a poor antibody level, revealing that improved vaccine response was reached with the highest dose tested, which is identical concentration of μg/serotypes used in other studies with BALB/C mice (33). Both single dose and two doses of pneumococcal vaccine were checked for antibody levels. Because response was higher with the two dose strategy (data not shown), this vaccine strategy was used for the following vaccine studies. Results were normalized with an internal standard as described in Animals and Methods. Our results showed that in WT mice, IgM antibody level increased with time following vaccination with PPV23 (2-way ANOVA for time: *p* < 0.001 and strains: *p* < 0.05) and PCV13 (2-way ANOVA for time: *p* < 0.001 and strains: *p* < 0.01) and was significantly above the initial concentration at day 21 for PPV23 (Bonferroni following two-way ANOVA; d21 *vs*. d0: *p* < 0.05) and day 28 (Bonferroni following two-way ANOVA; d28 *vs*. d0: *p* < 0.001) for PCV13. Conversely, *Ghrh*^−/−^ mice were unable to trigger a specific IgM response even after a second vaccination (Figures 1B,C).

### GH restores the IgM response of *Ghrh^−/−^* mice to PPV23 but not to PCV13

To assess whether GH treatment may restore vaccine immune response, we repeated the previous experimental protocol with supplementation by hGH (0 μg/g or 2 μg/g) to *Ghrh*^−/−^ mice (Figure 1A). Five-week hGH treatment partially restored IgM immune response to PPV23 (Bonferroni following two-way ANOVA; d35 *vs*. d0: *p* < 0.001) but only marginally to PCV13 (Bonferroni following two-way ANOVA; d28: 7.4 *vs*. 0%; GH 2 *vs*. 0 μg/g: *p* < 0.001; Figures 1B,C).

### *Ghrh^−/−^* mice exhibit a dramatic susceptibility to *S. pneumoniae* infection

**Figure 2:**
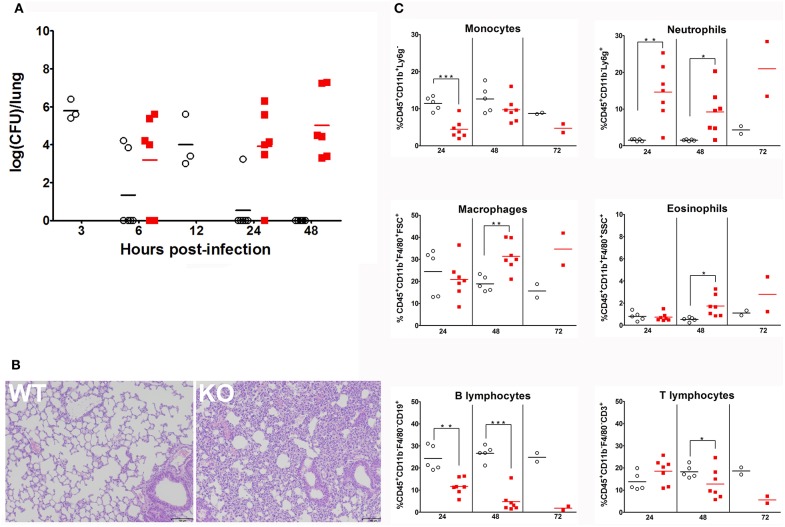
*Ghrh*^−/−^ mice fail to clear *S. pneumoniae* infection from lung **(A)** WT (◦) and KO mice (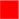) were inoculated by *S. pneumoniae* via i.n. At 3, 6, 12, 24, and 48 h post-infection, animals were sacrified and lung were sampled to measure colony-forming unit (CFU). All results are presented as individual response and mean. *N* = 3–6 mice per group. **(B)** Lung sections of *S. pneumoniae*-infected WT and KO mice HE-stained. Result shown are representative of two independent experiments. *n* = 6 mice per group. **(C)** Percentage of lung leucocytes: monocytes, neutrophils, macrophages, eosinophils, B lymphocytes and T lymphocytes of CD45.2 positive cells recruited to the lung of WT (◦) and KO (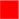) mice 24, 48 and 72 h post-infection. All results are presented as individual and mean response. *N* = 2–7 mice per group. *p < 0.05; **p < 0.001; ***p < 0.0001 (unpaired Student *t*-test).

The infection protocol consisted in administration of a non-lethal dose of *S. pneumoniae* (28). Mice were inoculated i.n. with 30 μl of 4 × 10^4^ CFU of *S. pneumoniae* serotype 1 strain E1586; enumeration of *S. pneumoniae* CFU in lung and spleen homogenates were compared between WT and KO at 6, 24, and 48 h post-infection. Absence of pre-existing oropharyngeal flora was demonstrated by i.n. inoculation with excipient alone. At 6 h following infection, there was no difference in lung bacterial CFU between the two strains. At 24 h post-infection, WT mice completely eliminated the infection, while KO mice failed to clear it and reached a high bacterial load level at 48 h post-infection (Figure 2A). Histological sections of lungs 24 h post-infection showed a massive infiltration of inflammatory cells and significant alterations of bronchoalveolar epithelium in KO lungs compared to WT (Figure 2B). Bacteria were not detected at any time in spleen homogenates from both WT and HZ mice, whereas KO mice developed a bacteremia 24 h post-infection and all reached death limit point 72 h post-infection (Table 1 and Supplementary Table 2). These data evidence that *Ghrh*^−/−^ mice have a dramatic susceptibility to *S. pneumoniae* infection, being unable to control a non-lethal infection dose.

**Table 1:** Bacteremia of WT, KO, and HZ mice in 6, 24, and 48 h post-infection.

	**WT**	**KO**	**HZ**
6 h	0/7 (*n* = 3M 4F)	0/7 (*n* = 2M 5F)	0/3 (*n* = 1M 2F)
24 h	0/13 (*n* = 5M 8F)	8/12^**^ (*n* = 4M 8F)	0/6 (*n* = 3M 3F)
48 h	0/13 (*n* = 7M 6F)	12/13^***^ (*n* = 7M 6F)	0/6 (*n* = 4M 2F)
72 h	0/3 (*n* = 1M 2F)	3/3 (*n* = 2M 1F)	0/3 (*n* = 3M 0F)

*The results are represented as ratio of bacteremic mice/total number of infected mice. Fisher's exact test*.

*** *p < 0.001*,

** *p < 0.005. M, male; F, female*.

### *Ghrh^−/−^* mice exhibit an increase in lung neutrophils, eosinophils, and a decrease in B lymphocyte percentage

We decided to investigate effects of this *S. pneumoniae* infection on immune responses in lungs. To this end, we designed a flow cytometry panel with a gating strategy that allowed us to determine the percentage of immune cells (macrophages, monocytes, neutrophils, eosinophils, B and T cells) that are known as major key cellular players in *S. pneumoniae* pulmonary infection (Supplementary Figure 1). FACS revealed that *Ghrh*^−/−^ monocytes 24 h post-infection were lower than in WT, while monocytes observed at 48 h post-infection in KO mice increased compared to 24 h (unpaired *t*-test; KO 48 vs. 24 h: *p* < 0.01) to reach a similar level as in WT. The percentage of neutrophils was larger during all infection period in KO compared to WT mice. In KO mice, macrophages and eosinophils percentage increased with time (unpaired *t*-test; KO 48 h vs. 24 h: *p* < 0.05 for macrophages and eosinophils). KO macrophages therefore showed a significant increase compared to WT but only after 48 h, as eosinophils. On the contrary, a striking lower percentage of B cells was observed in KO mice at all time, while a decrease in the percentage of KO T lymphocytes was observed only after 48 h (Figure 2C).

### *Ghrh^−/−^* mice exhibit an increase in *CsF3, Cxcl2, Cxcl9*, and a decrease in *Il17a, Cd40* lung expression

**Figure 3:**
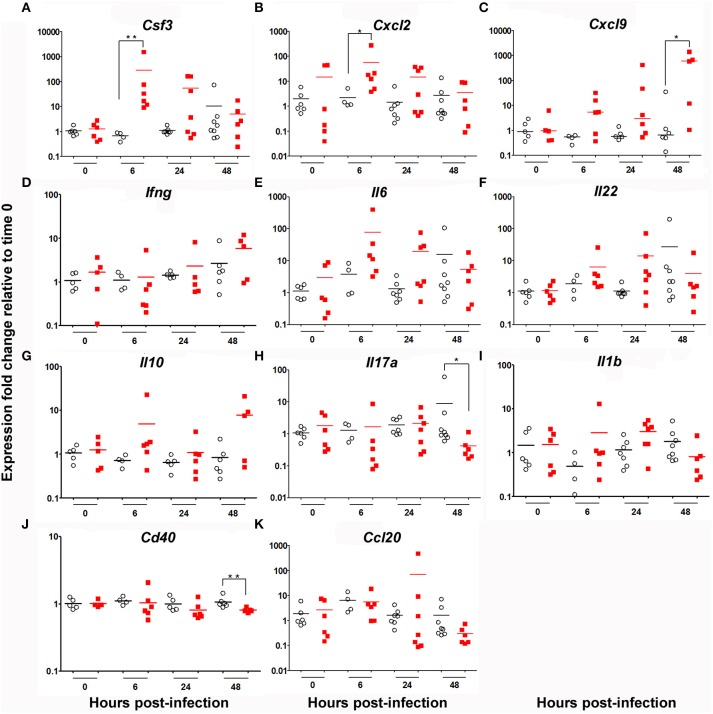
Cytokine mRNA signature in the lung of WT and KO mice after 6, 24 and 48 h infection by *S. pneumonia*. Messenger RNA expression of an array of cytokines, chemokines and receptors: **(A)** *Csf3*, **(B)** *Cxcl2*, **(C)** *Cxcl9*, **(D)** *Ifng*, **(E)** *Il6*, **(F)** *Il22*, **(G)** *Il10*, **(H)**, *Il17a* **(I)** *Il1b*, **(J)** *Cd40* and **(K)** *Ccl20* in the lung of WT (◦) and KO (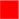) mice. *Hprt* was used as a house-keeping gene for analysis of *Cxcl9, Ifn*γ, *Il10* and *Cd40*. β*actin* was used as a house-keeping gene for analysis of *CsF3, Cxcl2, Il6, Il22, Ccl20, Il1*β and *Il17a*. Results are expressed as fold-change compared to their respective mock mice and are presented as individual and mean response. *N* = 3-9 mice per group. *p < 0.05, **p < 0.01 (Mann-Whitney *U*-test).

Expression of an array of cytokines (CSF3 [G-CSF], IFNγ, IL-1β, IL-6, IL-22, IL-17A, and IL-10), chemokines (CCL20, CXCL2 and CXCL9) and receptors (CD40) known to be involved in the regulation of *S. pneumoniae* inflammatory response (26) was measured at 0, 6, 24, and 48 h post-infection. Our results show a statistically significant increase in the expression of CSF3 and CXCL2 in KO compared to WT mice at 6 h post-infection (Figure 3). The transcripts specific for CXCL9 were also significantly higher in lung of KO mice 48 h post-infection. There was a non-significant elevation in the expression of mRNA for IFNγ, IL-6, IL-22, IL-10, CCL20, and IL-1β in KO compared to WT mice. Interestingly, expression of genes encoding IL-17A and CD40 were downregulated in KO compared to WT mice. These results are consistent with FACS experiments (Figure 2C) and concord with the action of G-CSF and CXCL2 in neutrophil recruitment (34, 35) as well as CXCL9 in macrophage and eosinophil infiltration (36).

### *Ghrh^−/−^* mice exhibit a specific profile of sera inflammatory proteins

Basal serum level of α-antitrypsin and IgA were higher in KO than in WT mice, while basal transferrin level was lower. During infection, α-antitrypsin and IgA remained higher and transferrin lower in *Ghrh*^−/−^ animals. Only at 6 h post-infection, the expression level of albumin increased significantly in WT mice (unpaired *t*-test; WT 0 vs. 6 h: *p* < 0.05), while expression level of albumin increased significantly 24 h post-infection in KO mice (unpaired *t*-test; KO 0 vs. 24 h: *p* < 0.05); at 48 h post-infection, both mice returned to basal level before infection. Regarding complement component C3 (C3), while there was no difference at basal level, the expression level of C3 increased significantly more in KO mice 6 h post-infection than in WT mice (Supplementary Figure 2). The expression level of C3 increased over time in *Ghrh*^−/−^ mice, and reached a significantly higher level at 48 h post-infection (unpaired *t*-test; KO 0 vs. 48 h: *p* < 0.05). An increase of the expression level of C3 was also seen in WT animals, but this elevation did not reach statistical significance. No differences were observed in total CRP and IgM, either in basal or infected conditions.

### *Ghrh^−/−^* B cell lymphopoiesis is not impaired compared to WT mice

**Figure 4:**
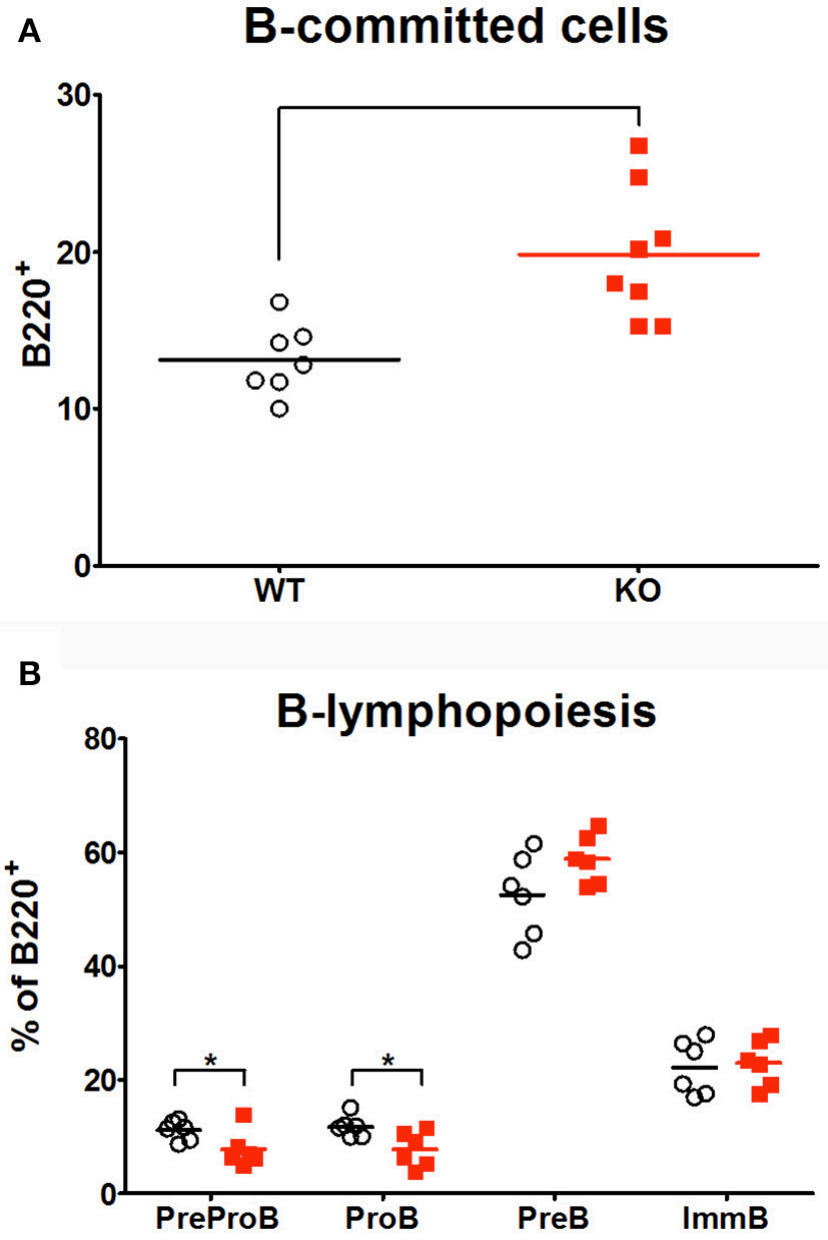
B lymphopoiesis. The percentage of B-committed cells amongst living cells **(A)** and the proportion amongst B220^+^ population of the four developmental stages of B cell development **(B)** were analyzed by flow cytometry in bone marrow of WT (◦), KO (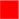) mice. Data (mean) are representative of 3 independent experiments. Unpaired *t*-test was used for statistical analysis. *N* = 7–8 per group. *p < 0.05.

In order to analyze if peripheral B cell defect observed in *Ghrh*^−/−^ mice resulted from a problem in B cell development in bone marrow, B lymphopoiesis was investigated by flow cytometry. B lineage committed cells expressed B220. Four developmental stages were distinguishable (Supplementary Figure 3): PreProB (CD43^+^ CD19^−^ IgM^−^), ProB (CD43^+^ CD19^+^ IgM^−^), PreB (CD43^−^ CD19^+^ IgM^−^) and immature B cells (CD19^+^IgM^+^). As shown in Figure 4, KO mice had an almost 2-fold higher proportion of B-committed B220^+^ cells compared to WT animals. In addition, the percentage of PreProB and ProB was significantly reduced in KO compared to WT while the percentage of PreB tended to increase (*p* = 0.0611). However, immature B proportion was similar between the three groups. Therefore, B lymphopoiesis seemed to be not deficient, and a defective lymphopoiesis could not explain decreased proportion observed in peripheral B cells. We can therefore conclude that *Ghrh*^−/−^ mice exhibit functional B lymphopoiesis and their bone marrow contains more B committed B220^+^ cells than normal mice.

### *Ghrh^−/−^* mice exhibit a low percentage of macrophages, monocytes, B lymphocytes but a high percentage of T lymphocytes

**Figure 5:**
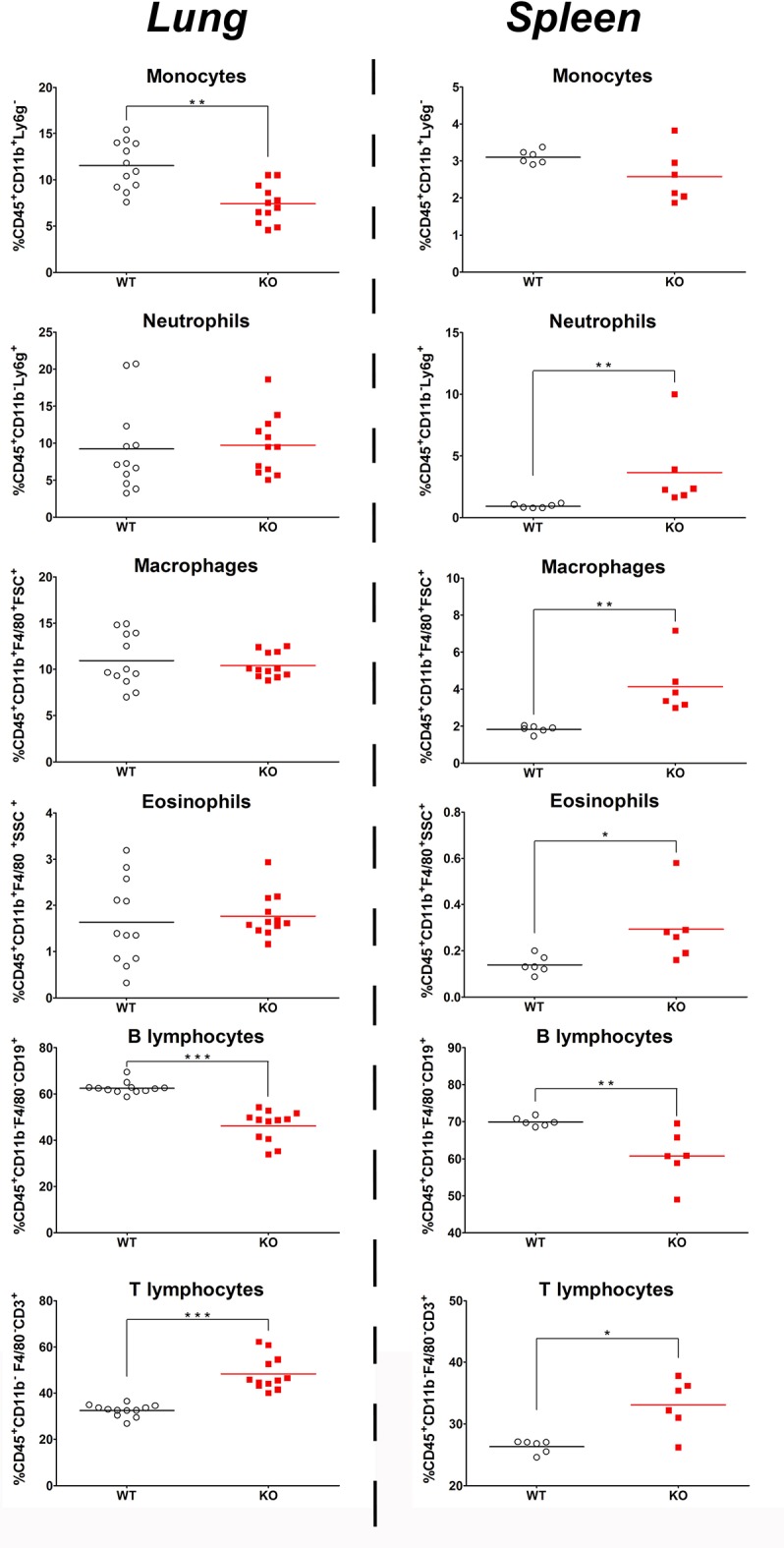
Basal percentage of immune cell in the lungs and spleen of KO and WT mice. Percentage of immune cells: monocytes, neutrophils, macrophages, eosinophils, B lymphocytes, and T lymphocytes in CD45.2 positive cells was measured in the lung **(left)** and spleen **(right)** of WT (◦) and KO (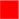) mice. All results are presented as individual and mean response and repeated as 3 independent experiments. *N* = 5–12 mice per group. *p < 0.05, **p < 0.01 and ***p < 0.01 (unpaired Student *t*-test).

In basal conditions, FACS data indicated a lower percentage of lung macrophages, monocytes and B lymphocytes in KO mice, while the percentage of T lymphocytes was higher compared to WT mice. There were no difference in the percentage of lung neutrophils, macrophages and eosinophils (Figure 5 left). These data indicated a similar distribution of granulocytes in lung of both mice strains, while monocytes and B lymphocytes exhibited a lower percentage and T lymphocytes a higher percentage in KO compared to WT mice.

The spleen also revealed a significant decrease in B lymphocyte and increase in T lymphocyte percentage (Figure 5 right). However and contrary to the situation in lungs, splenic granulocytes and macrophages were in higher proportion in KO than in WT while splenic monocytes were in similar proportion in both strains.

### *Ghrh^−/−^* mice exhibit a low percentage of splenic marginal and follicular B cells

**Figure 6:**
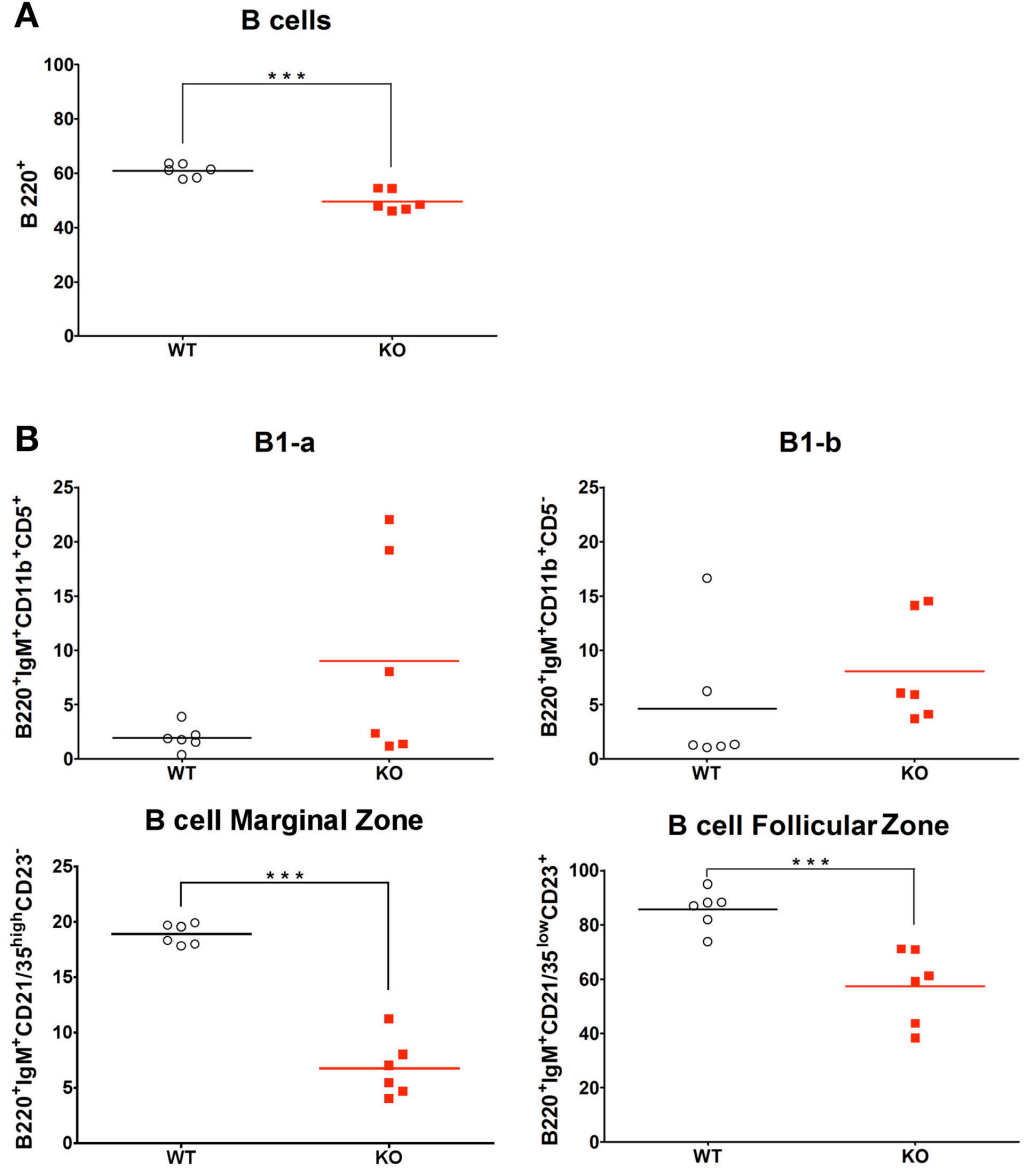
Splenic percentage of marginal and follicular zone B cells in WT and KO mice. Percentage of total B cells **(A)** and their subtypes **(B)**: B1-a, B1-b, marginal zone B cells and follicular zone B cells in the spleen of WT (◦) and KO (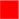) mice in basal conditions. All results are presented as individual and mean response. *N* = 6 mice per group. ***p < 0.0001 (unpaired Student *t*-test).

The spleen, in particular its MZ, plays a crucial role in screening and clearance of blood-borne antigens, such as *S. pneumoniae* (37, 38). To examine if splenic B cells might be responsible for differential responses to vaccination and infection, we checked the proportion of immune and B cell subtypes that are present in spleen in basal conditions. We designed a flow cytometry panel with a gating strategy that allowed us to identify different B cell subtypes in spleen of KO and WT mice (Supplementary Figure 4). By FACS analysis, B220 labeling confirmed the lower percentage of CD19 B lymphocytes in KO mice compared to WT mice (Figure 6). Noteworthy, this result was similarly seen in lung (Figure 5) as observed in blood and lymph nodes (14). Among different types of B cells, the percentage of MZ and FO B cells was decreased in KO in comparison with WT mice (Figure 6). Despite elevation in the percentage of B1-a and B1-b in KO mice in comparison to WT, the difference observed in these B cell subsets was not significant, maybe due to extensive proportion of mouse-to-mouse variation, B1-a (Mean ± SEM of WT 1.90 ± 0.464 *N* = 6, Mean ± SEM of KO 9.00 ± 3.827 *N* = 6) and B1-b (Mean ± SEM of WT 4.58 ± 2.541 *N* = 6, Mean ± SEM of KO 8.05 ± 2.015 *N* = 6).

### *Ghrh^−/−^* mice show a smaller, discontinuous and diffuse distribution of MARCO during *S. pneumoniae* infection

**Figure 7:**
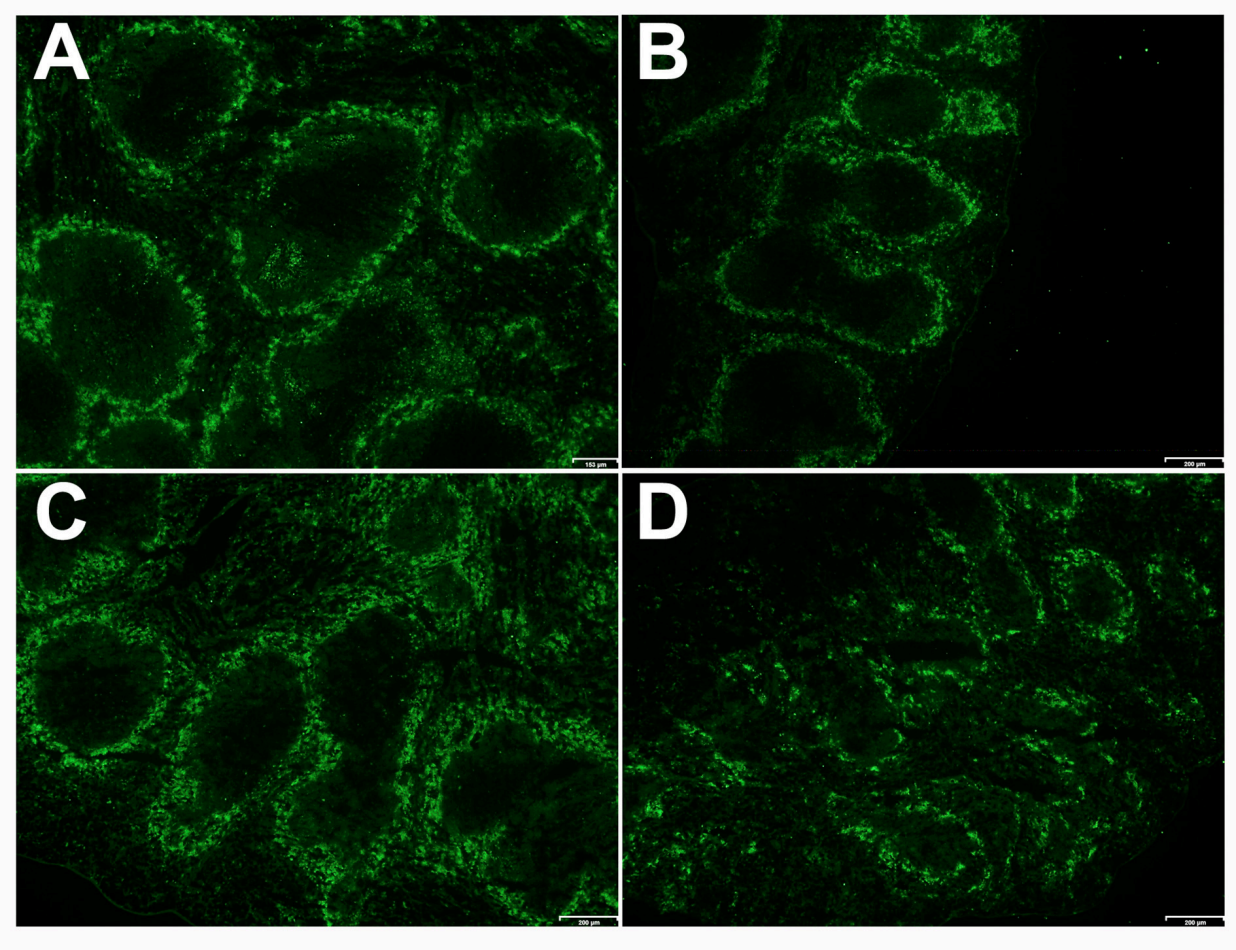
Spleen of KO and WT mice in mock and infected conditions. Immunofluorescence of the spleen sections of WT **(A,C)** and KO **(B,D)** mice 48 h after inoculation with DPBS **(A,B)** or *S. pneumoniae* **(C,D)** stained with anti-MARCO (green). Result shown is representative of 2 independent experiments.

We checked the distribution of a special subset of macrophage, which is a dominant population in MZ, and marked by Macrophage Receptor with Collagenous Structure (MARCO). This receptor is known to be expressed on macrophage subsets involved in clearance of *S. pneumoniae* infection (39). These macrophages are localized at the limit of MZ and FO. Both B cells and MZ macrophages are necessary for the integrity and normal activity of the MZ as demonstrated by the absence of MZ macrophages and Mucosal Addressin Cellular Adhesion Molecule-1 (MAdCAM-1) positive lining cells in the MZ sinus when B cells are missing during ontogeny (40). Importantly, it was shown that MARCO exhibit activity for endogenous ligands beared by MZ B cells and was related to retention of B cells in the MZ (41). MARCO staining demonstrated a continuous and organized distribution in splenic MZ in both WT and KO mice (in basal conditions). When WT mice were infected with *S. pneumoniae*, they conserved the same organized and continuous architecture of the spleen. However, infected *Ghrh*^−/−^ mice exhibited a dispersed MARCO distribution in the MZ (Figure 7), which can be related to the failure of bacterial clearance and bacteremia development in *Ghrh*^−/−^ mice when they are infected by a sublethal dose with *S. pneumoniae* (Table 1).

### *Ghrh^−/−^* mice are resistant to H1N1 infection

**Figure 8:**
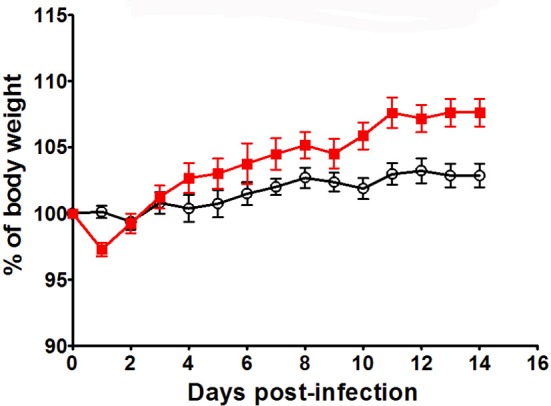
H1N1 infection of KO and WT mice. Body weight of WT (◦) and KO (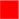) mice was measured after H1N1 infection, for 14 days by using sublethal dose (9pfu). Results are presented as mean ± SEM. N = 7 mice per group.

In order to verify whether KO mice have a general susceptibility to pathogens, we infected these mice with a thymus-dependent antigen: the H1N1 murine influenza virus. Infection follow-up was based on measurement of body weight percentage related to day 0. Body weight and behavior represent global health and state of the mouse during infection. These parameters are used to follow up susceptibility/resistance profile of animal model after infection (42). Mice were monitored daily for sign of illness and morbidity. Infected animal exhibit a restrained mobility from day 4 to day 10, associated with a rapid respiration and temperature diminish that reach the lowest level at day 10. No difference in body weight was observed between WT and KO mice by using a non-lethal dose [9 plaque-forming unit **(**PFU)] till day 10 and KO even gained more weight after that (2-way ANOVA for time and strains: *p* < 0.001 for time, *p* < 0.05 for strains; Bonferronni post-test *p* < 0.05 for KO vs. WT at day 10, 11, 12, 13, and 14) (Figure 8). This infectious dose did not affect differently body growth of WT and KO mice. In the context of a supra-lethal H1N1 infection (35 PFU), both strains reached death limit point (>3, Supplementary Table 1) at day 6 for *Ghrh*^−/−^ mice or day 8 for WT mice (data not shown).

Overall, these results suggest that *Ghrh*^−/−^ mice have a susceptibility to specific pathogens such as the thymus-independent antigen *S. pneumoniae*, rather than influenza virus (a thymus-dependent antigen).

## Discussion

The *Ghrh*^−/−^ mouse model presents a dwarf phenotype due to a severe deficiency of somatotrope GHRH/GH/IGF-1 axis. Lack of GHRH does not directly affect metabolism or reproduction, prolactin production is normal, and GH treatment is able to restore all somatic growth parameters (13, 43). These mice are less prone to develop experimental autoimmune encephalomyelitis (EAE) and GH supplementation (but not GHRH) restores original susceptibility of EAE (44). Parameters and T cell responses of *Ghrh*^−/−^ mice are not altered, but a relative B-cell lymphopenia and a severe splenic atrophy are constantly observed (14). Therefore, this study investigated vaccinal and anti-infectious responses of *Ghrh*^−/−^ mice to *S. pneumoniae*.

Our results show a significant increase in antibody levels over time especially after the second vaccination for WT mice, while KO mice were unable to trigger a specific IgM vaccine response (Figure 1). Remarkably, hGH supplementation for 5 weeks could partially restore pneumococcal IgM immune response to PPV23, but only marginally to PCV13 of *Ghrh*^−/−^ animals. This result suggests that hGH could exert an impact upon the splenic MZ rather than the FO, since PPV23 (and not PCV13) is involved in the stimulation of the MZ region (45). HGH was used to supplement *Ghrh*^−/−^ animals before vaccination. An action through the prolactin receptor could be involved since hGH has the ability to bind the prolactin receptor in humans, but also in mice (46). Nevertheless, as the prolactin production is not impaired in our *Ghrh*^−/−^ mice (13) and mouse GH do not bind the mouse prolactin receptor, the lack of response to vaccine appears to be related to GH through GH receptor and a binding of hGH to mouse prolactin receptor could only have a surrogate effect on response restoration.

In marked contrast to WT and HZ animals, KO mice were highly susceptible to a sublethal *S. pneumoniae* infection with 4 × 10^4^ CFU, as they failed to clear infection from lung, developed a bacteremia at 24 h, and had a survival limit of 72 h post-infection. *Ghrh*^−/−^ lungs 24 h post infection showed a massive infiltration of inflammatory cells when compared to WT lungs. FACS analyses showed that i.n. non-lethal dose of pneumococcus promoted a stronger innate immune response in KO compared to WT mice, as characterized by an early influx of inflammatory cells in lungs, predominantly neutrophils over all the times of infection, and macrophages and monocytes 48 h post-infection. These data suggest that the histological damages observed in lungs of infected *Ghrh*^−/−^ mice are related to neutrophil invasion as indicated by others (47, 48). Interestingly, the percentage of B lymphocytes decreased at all the times of infection and reached its lowest percentage 48 h post-infection in KO mice, while the percentage of T lymphocyte only decreased at 48 h post-infection, and there was a large percentage of mouse-to-mouse variability compared to WT mice.

As supported by RT-qPCR assays, infected *Ghrh*^−/−^ mice developped a strong and sustained innate immune response. In contrast, sublethal infection was not triggering any substantial proinflammtory responses in WT mice. We observed a clear signature of up regulated CSF3, as well as CXCL2 6 h post-infection genes in *Ghrh*^−/−^. We also observed a significant increase in the expression of CXCL9 48 h post-infection compared to WT mice. However, we observed an increased expression of IL-17A and CD40 in lung homogenates of WT in comparison with *Ghrh*^−/−^ mice 48 h post-infection. These data are in agreement with FACS experiments since CSF3 and CXCL2 are involved in neutrophil recruitment (34, 35) and CXCL9, a ligand of CXCR3 expressed by macrophages, neutrophils, epithelial and endothelial cells in lungs (36), exhibits the ability to kill pneumococcus *in vitro* (49). Moreover, the decrease in IL-17A and CD40 is consistent with the decrease observed in percentage of T and B lymphocytes at the same time point, as IL-17A is a pro-inflammatory cytokine produced by activated T cells (50) and CD40 is a co-stimulatory protein found on antigen-presenting cells firstly characterized on B cells, that could be associated with induced humoral response against pneumococcus (51). Interestingly, it has been reported that IL-17A plays a crucial role against development of *S. pneumoniae* colonization, while it can also be deleterious in face of some invasive pneumococcal strains (52, 53). The absence of an increase in IL-17A expression in infected *Ghrh*^−/−^ mice could therefore be a key to explain their dramatic susceptibility to the pathogen.

Based on strong inflammatory response observed in *Ghrh*^−/−^ mice, we decided to study the serum level for an array of acute phase proteins that are regulated in response to inflammation (C-reactive protein, Complement component C3, IgM and IgA). Serum CRP and C3 were measured because of their close interaction and their important role in regulating *S. pneumoniae* infection. The activation of classical complement pathways, such as the C3 complement component, is driven by CRP, resulting in opsonisation of pneumococcus (54) and in limiting rapid bacteremia during pneumococcal infection (25). Our results show a similar expression level of CRP and C3 in both mice strains prior to infection, as well as during the following time points post-infection, suggesting a similar capacity to activate the classical complement pathway. Additionally, levels of serum IgM and IgA were measured because of their essential role in resistance against *S. pneumoniae*: IgM protects against invasive pneumococcal infection and IgA is necessary against pneumococcal colonization (55, 56). Our result show a comparable basal level of IgM, as well as during each of the infection time points, between WT and KO mice, suggesting a normal capacity to induce an IgM response prior and during infection. However, this result could suggest that *Ghrh*^−/−^ mice are not able to produce natural specific IgM to pneumococcal serotype as supported by vaccine results against *S. pneumoniae* serotype 1.

Overall, these data demonstrate that *Ghrh*^−/−^ mice are more susceptible to a sublethal *S. pneumoniae* dose, and develop an inflammatory immune response stronger than WT, despite similar expression profile of acute phase proteins. To gain further insight into this difference, we investigated the proportion of immune cells involved in immune response to *S. pneumoniae* that are present in lung in basal conditions. By FACS analysis, we observed a lower percentage of macrophages, monocytes and B lymphocytes in *Ghrh*^−/−^ mice, while the percentage of T lymphocytes was higher. The innate immune response involved in clearance of pneumococcal lung infection is driven by resident cells, since alveolar macrophages prompt an inflammatory response by releasing pro-inflammatory cytokines covering IL-6, IL-1β and other chemokines that recruit additional immune cells involved in microbial clearance, such as neutrophils (57). The low percentage of macrophages in lung of *Ghrh*^−/−^ mice could cause inability to initiate a strong early inflammatory response, as suggested by the lack of significant increase in IL-6 and IL-1β expression. Remarkably, the lower percentage of B lymphocytes and the higher percentage of T lymphocytes in KO compared to WT mice was also observed in blood, lymph node and spleen (14).

Based on the B/T lymphocyte imbalance, as well as results obtained from vaccine and infectious response against *S. peumoniae*, we decided to investigate the spleen of *Ghrh*^−/−^, as it is widely accepted that this organ plays a pivotal role in pneumococcus clearance as shown by the fact that asplenic individuals and mice have an increase susceptibility and a limited capacity to clear *S. pneumoniae* infection (58, 59). The spleen filters blood and captures pathogens, especially encapsulated bacteria (60). Protection is provided by antibodies against TI-2 antigens provided by macrophages and B cells MZ (61). The anatomy of the spleen is divided into 2 regions: the MZ that directly interacts with the antigens of the blood stream and is primarily involved in the T cell independent responses (62) and the FO that is in close proximity to T cell zones and is implicated in T cell dependent immune responses (63). Splenic B cells are divided into B1 and B2 cells. B1 cells are sub-classified into B1-a and B1-b (64), while B2 are sub-classified into MZ and FO (65). B1 and MZ B cells generate early and rapid humoral response by producing IgM or IgG3 (66). FO cells allow an isotype switching to IgG by cooperation with T cell regions and this process requires days to weeks to be established (63). We compared the proportion of different immune cells as well as the proportion of B cell subsets that are present in the spleen in basal conditions. While B cell lymphopoiesis was not impaired in *Ghrh*^−/−^ bone marrow, we observed a lower percentage of B lymphocytes in the spleen of *Ghrh*^−/−^ mice, while the percentage of T lymphocytes was higher compared to WT mice, similarly to FACS analysis in lungs. In contrast, we observed a higher percentage in the proportion of neutrophils, macrophages and eosinophils in KO compared to WT mice. Regarding B cell subtypes, we observed a decrease in the percentage in MZ and FO among the different types of B cells in KO in comparison with WT mice. These data can explain why *Ghrh*^−/−^ mice are unable to trigger a specific vaccine immune response to *S. pneumoniae*, and could be one of the principal causes of susceptibility observed by using a sublethal *S. pneumoniae* infection dose. These results led us to examine expression of MARCO in the spleen, since Chen *et al*. previously observed that ablation of MARCO results in an impaired vaccine response against *S. pneumoniae* and modify the percentage and the distribution of macrophages that are present on the spleen MZ (67).

By examining histological sections of spleen in basal conditions, our results showed a continuous and organized distribution of MARCO in the spleen MZ of WT and KO mice in basal conditions, in contrast to what was reported in MARCO KO mice. However, by examining histological sections of infected mice spleen, we observed a dispersed MARCO distribution in the MZ of *Ghrh*^−/−^ mice 24 h post-infection, whereas WT mice still show a continuous and organized distribution of MARCO. These results can suggest that cooperation between B and MARCO cells is similar in KO and WT mice before infection, but is altered by the bacteremia development in *Ghrh*^−/−^ mice as the cooperation between B cells and macrophages is essential for appropriate organization of the MZ, as well as for function and contact between these immune cells (68).

It was important to assess if *Ghrh*^−/−^ mice have a general susceptibility to all pathogens. It was previously reported that hypophysectomized rats were more sensible to *Salmonella thyphimurium* infection, and that GH supplementation could partially restore their resistance (69). For this purpose, we decided to infect *Ghrh*^−/−^ mice with a T cell dependent antigen, murine influenza virus H1N1. The susceptibility of *Ghrh*^−/−^ mice to H1N1 was evaluated using a sublethal dose in C57BL/6 mice (31). Our results showed no difference in body weight changes between WT and KO mice. These results suggest that *Ghrh*^−/−^ mice have a specific susceptibility to pathogens such as encapsulated thymus-independent *S. pneumoniae*.

In conclusion, *Ghrh*^−/−^ mice exhibit a severe splenic atrophy and B-cell lymphopenia in lung and spleen. Moreover, these mice do not elicit a response to pneumococcal vaccines, possibly explained by a low level of B cells in the spleen MZ and FO. GH treatment restores vaccine response to PPV23. These mice are extremely susceptible to a sublethal infection by *S. pneumoniae* and develop fatal bacteremia, probably related to massive neutrophil recruitment in the lung associated with the lack of IL-17A increase. Thus, these observations evidence that the somatotrope GHRH/GH/IGF-1 axis plays a crucial role in immunological defense against *S. pneumoniae*.

Isolated GH deficiency in human is quite rare, but its incidence is not trivial (70–73) and a higher incidence of death in female subjects between age 4-20 has been reported (74). Recently, low IgG has been evidenced in GH deficient subjects and a modification of immune response is suspected to be without clinical relevance in daily condition, but may contribute to an unfavorable outcome in front of more severe infections (75, 76). In future experiments, it will be interesting to investigate the precise impact of isolated GH deficiency on fetal development of the spleen, and to explore spleen development and pneumococcal responses in children with GH deficiency as it has been shown that GHRH receptor deficient human has reduced spleen (77). These observations could suggest pediatricians to monitor splenic development, vaccinal response, and susceptibility to *S. pneumoniae* in children with congenital (mainly) and acquired forms of GH deficiency.

## Author contributions

KF and GB are equal first authors and performed all experiments. CC-R assisted technically KF and GB. KF, GB, HM and VG designed most experimental protocols. CD designed exploration of lung immune cells. MM, YB and FB provided their expertise in infectious diseases. PM, PQ and J-CS provided their expertise in *S. pneumoniae* infection. A-SP provided her expertise about clinical defects of the somatotrope axis in children. DD provided his expertise in murine influenza virus infection. RS provided GIGA-I3 with Ghrh^−/−^ mice and corrected the manuscript. KF, VG and HM wrote the manuscript. VG and HM are equal last authors and supervised the whole work.

### Conflict of interest statement

The authors declare that the research was conducted in the absence of any commercial or financial relationships that could be construed as a potential conflict of interest.

## Abbreviations

BSA: Bovine serum albumin
C3: complement component C3
CFU: colony forming unit
DPBS: Dulbecco's phosphate-buffered saline
ELISA: Enzyme-linked immunosorbent assay
Ghrh: Growth hormone-releasing hormone
i.p: Intraperitoneal
KO: Knockout mice
OD: Optical density
Pnc: Pneumococcal
PnPS1: Pneumococcal polysaccharide serotype 1
PCV13: Prevnar 13
PPV23: Pneumovax 23
P/S: Penicillin and streptomycin
RT: Room temperature
RPMI: Roswell Park Memorial Institute medium
s.c: Subcutaneous
*S. pneumoniae*: *Streptococcus* pneumonia
THYB: Todd Hewitt Yeast Broth medium
V/V: Volume/volume
WT: Wild type mice
MZ: Marginal zone
FO: Follicular zone.
