# Supplementary material for: Growth Hormone (GH) Deficient Mice With GHRH Gene Ablation Are Severely Deficient in Vaccine and Immune Responses Against Streptococcus pneumoniae
Source: Front Immunol. 2018 Oct 2;9:2175. doi: 10.3389/fimmu.2018.02175 (PMC6176084; doi:10.3389/fimmu.2018.02175)
Supplement: Supplementary file 1 [file Table_1.pdf]

Supplementary Table 1 : Scoring of morbidity after infection

Mice were sacrified when reaching a score >3.

Score	**1**	**2**	**3**	**4**	**5**
Temperature	38	35	33	30	28
Weight (%)	100	90	85	80	< 80
Weakness	No		Moderate		Extented
Mobility	Normal		Moderate		None
Ruffled fur	No		Localised		Extented
Lordosis	No		Moderate		Yes
