# Supplementary material for: Growth Hormone (GH) Deficient Mice With GHRH Gene Ablation Are Severely Deficient in Vaccine and Immune Responses Against Streptococcus pneumoniae
Source: Front Immunol. 2018 Oct 2;9:2175. doi: 10.3389/fimmu.2018.02175 (PMC6176084; doi:10.3389/fimmu.2018.02175)
Supplement: Supplementary file 2 [file Table_2.pdf]

Supplementary Table 2: Forward and reverse primers for mouse transcripts coding *Csf3*, *Cxcl20*, *Cxcl9*, *Ifng*, *Il6*, *Il22*, *Il10*, *Ccl20*, *Il1b*, *Il17a*, *Cd40*, *Hprt* and *actinb*

Name	Forward sequences	Reverse sequences
Cd40	GACTCAGGTGAATTCTCAGC	GTGGCCATCTCCATAACTCC
Cxcl9	TGTGGAGTTCGAGGAACCCT	AGTCCGGATCTAGGCAGGTT
Ifng	CGGCACAGTCATTGAAAGCC	TGTCACCATCCTTTTGCCAGT
Il10	TCTTCAGTTCTCAGGACGCC	GCAATGAATTCTAGGCTCAGGC
Ccl20	TTTTGGGATGGAATTGGACAC	TGCAGGTGAAGCCTTCAACC
Cxcl2	CCCTCAACGGAAGAACCAAA	CACATCAGGTACGATCCAGGC
CsF3	CCTGGAGCAAGTGAGGAAGA	CAGCTTGTAGGTGGCACACA
Il1b	AATCTATACCTGTCCTGTGTA ATGAAAGACTGG	TGGGTATTGCTTGGGATCCA
Il17a	CTCCAGAAGGCCCTCAGACTAC	GGGTCTTCATTGCGGTGG
Il6	GTTCTCTGGGAAATCGTGGAAA	AAGTGCATCATCGTTGTTCATACA
Il22	ATACATCGTCAACCGCACCTTT	AGCCGGACATCTGTGTTGTTAT
Hprt	GATTAGCGATGATGAACCAGGTT	CCTCCCATCTCCTTCATGACA
Actb	GCTTCTTTGCAGCTTCTTTGT	CGTCATCCATGGCGAACTG
