# Supplementary material for: Growth Hormone (GH) Deficient Mice With GHRH Gene Ablation Are Severely Deficient in Vaccine and Immune Responses Against Streptococcus pneumoniae
Source: Front Immunol. 2018 Oct 2;9:2175. doi: 10.3389/fimmu.2018.02175 (PMC6176084; doi:10.3389/fimmu.2018.02175)
Supplement: Supplementary file 3 [file Image_1.pdf]

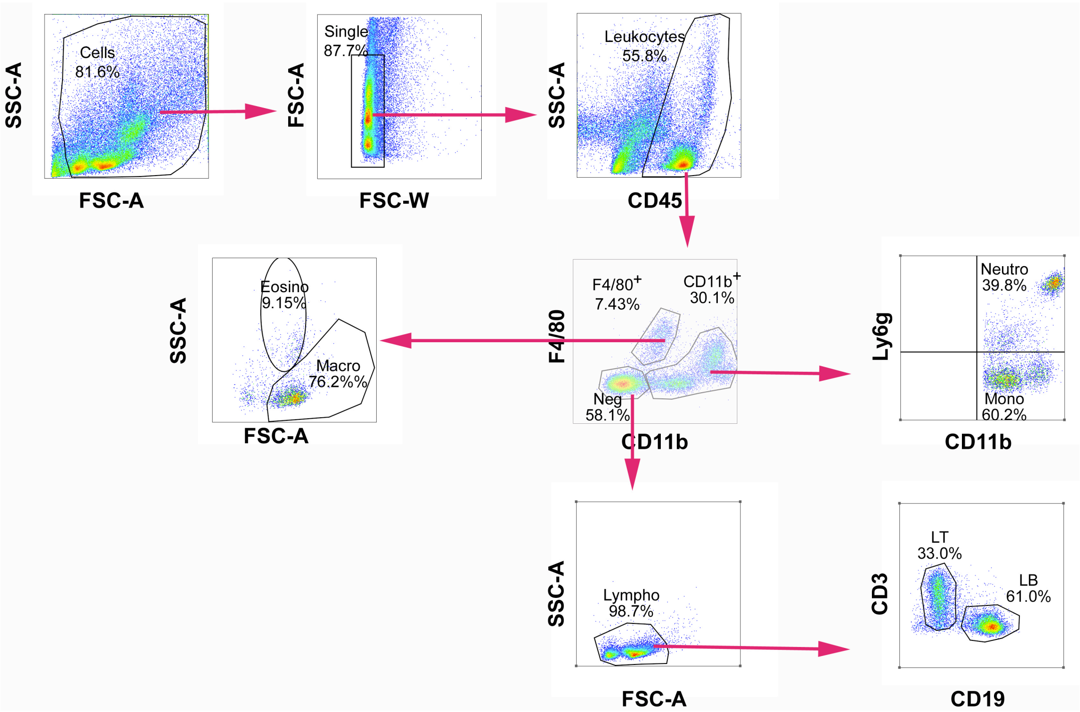

Supplementary figure 1: Gating strategy of immune cell selection by flow cytometry. Upper pannels : selection of single leucocytes with CD45 marker.

Middle pannels : selection of 4 types of leucocytes based on the expression of CD11-b and F4/80. The distinction between macrophages (FSC-A^{+}) and eosinophils (SSC-A^{+}) is based on their size. The distinction between monocytes and neutrophils is based on their ly6g expression, where the neutrophils are CD11b^{+} ly6g^{+} and the monocytes are CD11b^{+}ly6g^{-}.

Lower pannels : we consider that the CD11-b^{-} cells are lymphocytic cells after size verification. B cells (CD3^{-}CD19^{+}) and T cells (CD3^{+}CD19^{-}) are selected with CD19 and CD3 markers.
