# Supplementary material for: Growth Hormone (GH) Deficient Mice With GHRH Gene Ablation Are Severely Deficient in Vaccine and Immune Responses Against Streptococcus pneumoniae
Source: Front Immunol. 2018 Oct 2;9:2175. doi: 10.3389/fimmu.2018.02175 (PMC6176084; doi:10.3389/fimmu.2018.02175)
Supplement: Supplementary file 4 [file Image_2.pdf]

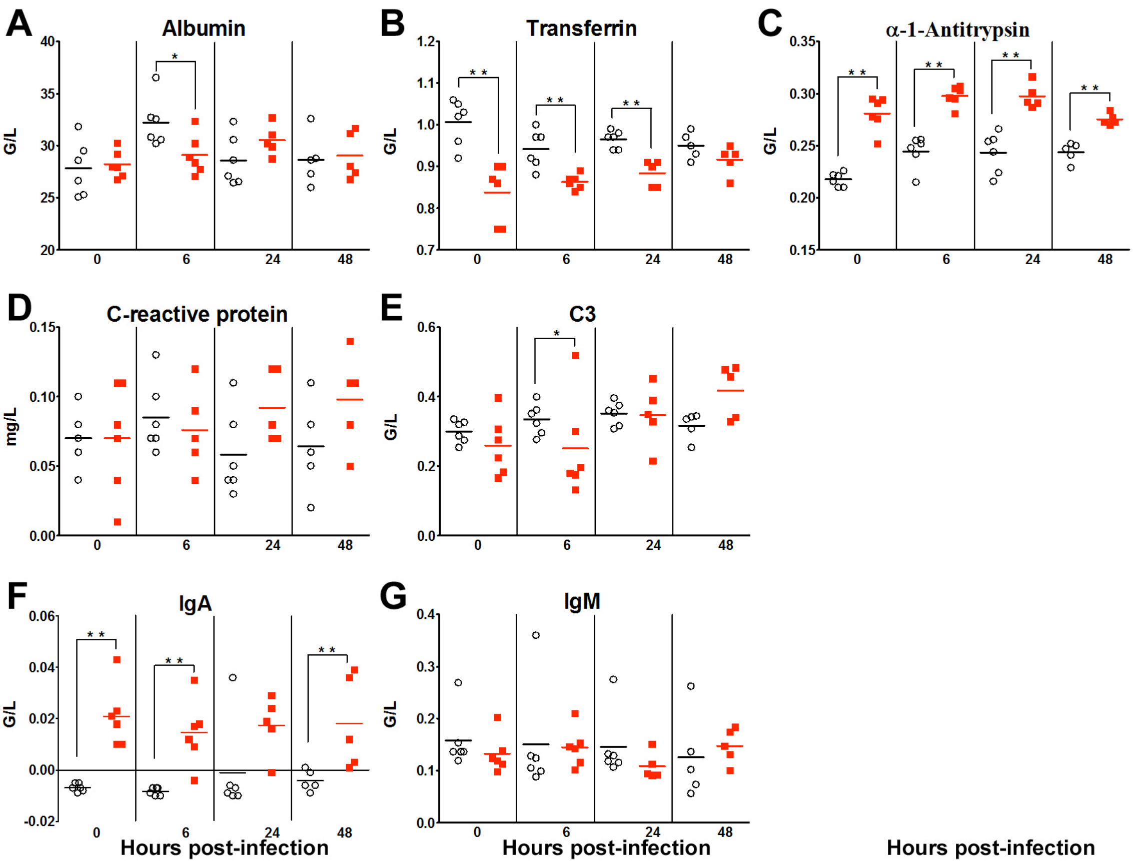

Supplemental figure 2: Sera protein level determination in ghrh$^{-/-}$ and WT mice. The concentration of sera proteins: **A**) albumin, **B**) transferrin, **C**) _-1-antitrypsin, **D**) C-reactive protein, **E**) C3 component complement, **F**) IgA antibodies, and **G**) IgM antibodies was measured in WT (_) and KO (_) mice at 0, 6, 24 and 48h post-*S. pneumoniae* infection. All results are presented as individual response and mean. n=5 mice per group. *, p_0.05; **, p_0.001.
