# Supplementary material for: Growth Hormone (GH) Deficient Mice With GHRH Gene Ablation Are Severely Deficient in Vaccine and Immune Responses Against Streptococcus pneumoniae
Source: Front Immunol. 2018 Oct 2;9:2175. doi: 10.3389/fimmu.2018.02175 (PMC6176084; doi:10.3389/fimmu.2018.02175)
Supplement: Supplementary file 5 [file Image_3.pdf]

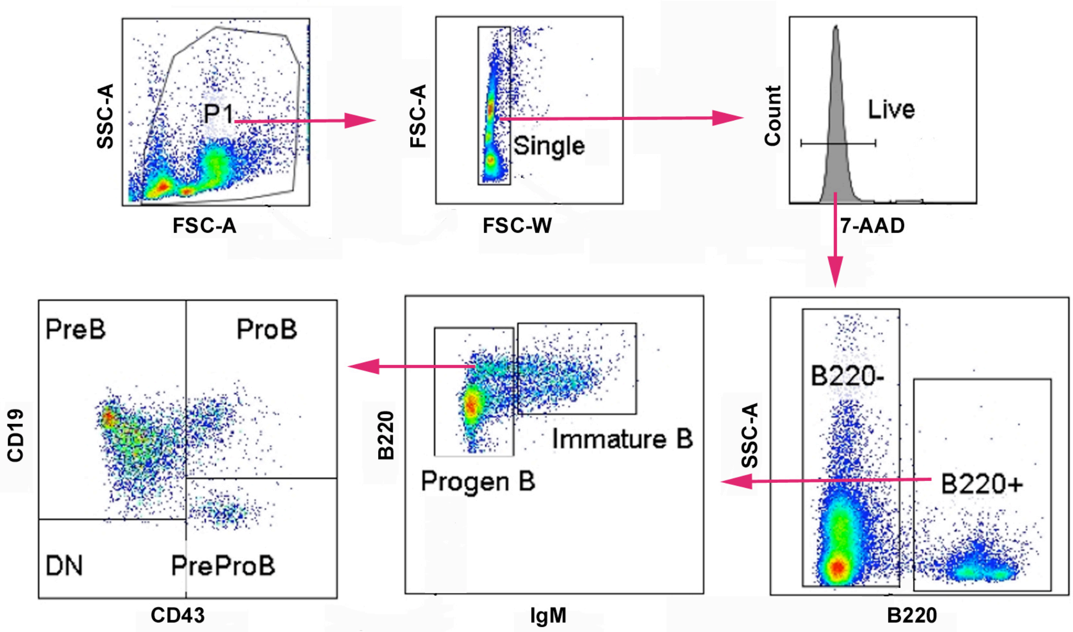

Supplemental Figure 3 : Gating strategy for flow cytometry of B lymphopoiesis in bone marrow. 500,000 cells were labeled and analyzed on FACS Verse. Debris and dead cells were excluded in function of their SSC-A/FSC-A profile (P1) and single cells were selected by FSC-A/FSC-W gating. 20,000 living cells (7-AAD negative) were recorded. B-lineage committed cells were gated as $B220^+$ cells. IgM expression distinguished immature B cells from earlier progenitors. Amongst IgM^- progenitors, PreProB cells ($CD43^+CD19^-$) evolved into ProB cells ($CD43^+CD19^+$) that further differentiate into PreB cells ($CD43^-CD19^+$).
