# Supplementary material for: Growth Hormone (GH) Deficient Mice With GHRH Gene Ablation Are Severely Deficient in Vaccine and Immune Responses Against Streptococcus pneumoniae
Source: Front Immunol. 2018 Oct 2;9:2175. doi: 10.3389/fimmu.2018.02175 (PMC6176084; doi:10.3389/fimmu.2018.02175)
Supplement: Supplementary file 6 [file Image_4.pdf]

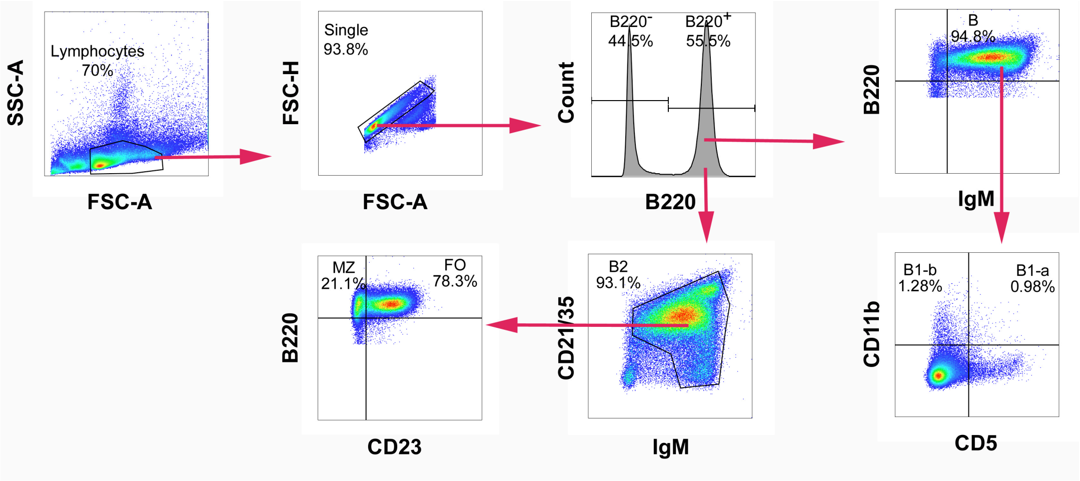

Supplementary Figure 4: Gating strategy of B cell subtype selection by flow cytometry. Upper pannels : selection of single leucocytes based on their size (FSC-A and SSC-A), single cells based on FSC-A and FSC-H, and selection of total B cells based on the expression of B220 and IgM markers.

Lower pannels : we used CD21/35 to select B2 cells and used CD23 to discriminate the MZ and FO cells. MZ cells are ($B220^{+}IgM^{+}CD21/35^{high}CD23^{-}$), FO cells are ($B220^{+}$ $IgM^{+}CD21/35^{low}CD23^{+}$). From B ($B220^{+}IgM^{+}$) cells, CD5 discriminates B1-A and B1-b : B1-a cells are $CD5^{+}$, B1-b cells are $CD5^{-}$.
